# PhenoPhyte: a flexible affordable method to quantify 2D phenotypes from imagery

**DOI:** 10.1186/1746-4811-8-45

**Published:** 2012-11-06

**Authors:** Jason M Green, Heidi Appel, Erin MacNeal Rehrig, Jaturon Harnsomburana, Jia-Fu Chang, Peter Balint-Kurti, Chi-Ren Shyu

**Affiliations:** 1 Department of Computer Science, University of Missouri, Columbia, MO, 65211, USA; 2 Division of Plant Sciences, University of Missouri, Columbia, MO, 65211, USA; 3 Biology/Chemistry Department, Fitchburg State University, Fitchburg, MA, 01420, USA; 4 Informatics Institute, University of Missouri, Columbia, MO, 65211, USA; 5 Department of Plant Pathology, North Carolina State University, Raleigh, NC, 27695, USA; 6 Informatics Institute & Department of Computer Science, University of Missouri, Columbia, MO, 65211, USA; 7 371 Bond Life Sciences Center, Columbia, MO, 65211, USA

**Keywords:** Herbivory, Pathogens, Genetic variation, Digital phenotyping

## Abstract

**Background:**

Accurate characterization of complex plant phenotypes is critical to assigning biological functions to genes through forward or reverse genetics. It can also be vital in determining the effect of a treatment, genotype, or environmental condition on plant growth or susceptibility to insects or pathogens. Although techniques for characterizing complex phenotypes have been developed, most are not cost effective or are too imprecise or subjective to reliably differentiate subtler differences in complex traits like growth, color change, or disease resistance.

**Results:**

We designed an inexpensive imaging protocol that facilitates automatic quantification of two-dimensional visual phenotypes using computer vision and image processing algorithms applied to standard digital images. The protocol allows for non-destructive imaging of plants in the laboratory and field and can be used in suboptimal imaging conditions due to automated color and scale normalization. We designed the web-based tool PhenoPhyte for processing images adhering to this protocol and demonstrate its ability to measure a variety of two-dimensional traits (such as growth, leaf area, and herbivory) using images from several species (*Arabidopsis thaliana* and *Brassica rapa*). We then provide a more complicated example for measuring disease resistance of *Zea mays* to Southern Leaf Blight.

**Conclusions:**

PhenoPhyte is a new cost-effective web-application for semi-automated quantification of two-dimensional traits from digital imagery using an easy imaging protocol. This tool’s usefulness is demonstrated for a variety of traits in multiple species. We show that digital phenotyping can reduce human subjectivity in trait quantification, thereby increasing accuracy and improving precision, which are crucial for differentiating and quantifying subtle phenotypic variation and understanding gene function and/or treatment effects.

## Background

Forward or traditional genetics is based on the identification of genetic variation associated with observable changes in phenotypic traits like growth rate, morphology, or coloring followed by the identification and characterization of the gene associated with the variation. In reverse genetics, the expression or sequence of a known gene is manipulated, and the resulting phenotypic response is measured. Since the phenotypic changes used in both approaches can be subtle, it is crucial that one has the ability to accurately and objectively characterize changes in phenotype to elucidate and understand gene function.

In some traits, phenotypic differences are easily seen as binary (yes/no) changes in survivorship or pigmentation [1]. While identifying yes/no phenotypes is relatively simple, it can be considerably more difficult to assess phenotypic variation that is measured on a continuous scale (i.e. quantitative variation). In these cases, phenotype quantification is often performed using rubrics. For example, pathogenesis or herbivory can be evaluated using scales based on infection or leaf damage levels assessed visually [2-4], which can introduce error into the measurements, especially if multiple people are scoring or if a presentation of the phenotype does not easily fit into one of the predefined categories. While rubrics are often the cheapest, fastest, and easiest method of quantifying these phenotypes, there are other methods of phenotypic measurement that involve harvesting all or portions of the plant. One can measure pathogenesis by grinding plant tissue and performing time-intensive serial dilutions to determine the number of Colony Forming Units (CFUs) of bacteria in each sample e.g. [5,6]. One can also subsample tissue over time for analysis of pathogens or herbivory; however, this can introduce an additional wounding treatment, which could render the plant unsuitable for measurements taken at a later time.

What is needed is an inexpensive, general, and flexible framework from which plant phenotypes can be accurately measured. The framework needs to be relatively simple and include the ability to measure a range of traits from a variety of species in many growing conditions. Ideally, one would also like the framework to accommodate phenotype measurement without being destructive to the plant and to allow repeated measurements of a given trait on the same plant over time.

There are several computational systems that meet some of the above criteria. LemnaTec’s Arabidopsis Morphological Phenotyping Assessment system (see http://www.lemnatec.com) images and analyzes a number of traits; however, this system is quite expensive and is not suitable for all situations (e.g. phenotyping in the field). Alternatively, ASSESS, an image analysis software for plant disease quantification [7], provides quick and automatic measurement of disease burden, area, ground cover, among other traits, but the software is not free, requires manual calibration unless connected to a scanner, and does not link repeated measurements on the same plant over time. Similarly, Iyer-Pascuzzi *et al*. developed an imaging platform and analysis application for measuring plant root systems [8] which nondestructively images plant root systems grown in cylinders and, with the use of a turntable mechanism, makes a 3D reconstruction of the root system. However, this method cannot be used for plants grown in soil or for above ground parts. Some applications exist for analyzing leaf shape, including the LeafAnalyser program, which primarily uses principal component analysis (PCA) to automatically determine leaf shape variation [9]; LAMINA, a tool that provides automatic measurement of a variety of characteristics related to leaf size and shape [10]; and LeafProcessor, an application that semi-automatically supplies a number of single-metric parameters and PCA analysis for leaf size and shape including contour bending energy [11]. While all three of these leaf shape analysis methods provide accurate and useful analysis applicable to leaves of various plant species, each has important limitations. For example, LeafAnalyser, LAMINA, and LeafProcessor require leaves to be removed from the plant to be imaged, making before and after images impossible to acquire. ImageJ, a popular utility available from the National Institutes of Health [12], is a powerful and extensible application that can be used to measure a variety of phenotypic traits captured by images. However, a nontrivial amount of image processing and programming background is required to determine the functions and parameters needed to obtain the desired phenotype measurements and to write a macro or script to automate the processing pipeline for batch processing. More recently, three platforms for measuring plant growth have been developed including GROWSCREEN [13], PHENOPSIS [14], and a three-dimensional growth phenotyping pipeline from LemnaTec [15]. While all three of these growth analysis methods provide highly accurate and sophisticated growth measurements, they utilize non-portable conveyor belts and/or robotic mechanisms to automate imaging. Unfortunately, plants grown in the field cannot be imaged this way and most research groups are unable to afford the equipment, maintenance, and space required by these systems. Last, none of the programs described above perform automatic normalization of images to control for variation in lighting and scale that arise naturally from different growth environments. See the article by Furbank and Tester [16] for a recent review of computational technologies available for plant phenotyping.

We report here a free software package that performs automated measurements of two-dimensional phenotypic traits suitable for many imaging applications, both destructive and nondestructive. The usefulness, flexibility, novelty, and applicability of the protocol and software are demonstrated with several example experiments.

## Results and discussion

### Phenophyte: the web application

PhenoPhyte is a new web-based application designed to automatically and efficiently measure area-related phenotypic traits from imagery and multiple experimental setups. The web application was designed for images of individual leaves (both detached and *in situ*) and rosettes, and permits imaging of multiple objects in a single frame. To reduce the pre-processing of phenotype trait data, PhenoPhyte also provides a means to track objects across images and provide measurements of temporally varying traits such as growth and herbivory.

The computational mechanisms underlying PhenoPhyte represent a unique combination of existing image processing techniques and novel algorithms. Existing algorithms chosen for their efficiency and accuracy, include Sobel and Canny edge detection, connected component labeling, and thresholding [17]. Novel algorithms include (1) a robust module for identifying the mini color checker in the image that is invariant to color checker orientation, insensitive to color distortion of the image, and quite insensitive to obstruction of the color checker, (2) a function to compute the appropriate transformation on each color channel to normalize the image spectrally, 3) image normalization based (1) and (2), (4) a component to track relative plant/pot positions across image sequences, and (5) linking of objects and their measurements across image sequences to calculate changes. See the Image Processing subsection under Methods for more detailed explanation of the algorithms.

Users are able to upload large amounts of imagery to the server for batch processing. Each upload can handle up to 2GB or 500 images, whichever comes first; however, if users have more images, they may repeat this process until the entire set is uploaded. Following processing, users are able to review the image results and make adjustments where necessary. Additionally, PhenoPhyte automatically generates graphs showing both experiment-wide and individual plant results. This may include growth curves, stacked bar graphs illustrating herbivory, or simple bar charts for area traits. Finally, the web application allows users to download image results (normalized images and segmentation results) and measured trait values in CSV format, which can be viewed directly in Microsoft Excel. This free web application can be found at http://PhenomicsWorld.org/PhenoPhyte. Source code is available by request as well. A detailed manual is available at the website to aid current and interested users in navigating the software.

### Image requirements and recommendations

PhenoPhyte supports robust and flexible phenotype expression quantification based on images that adhere to three simple requirements. First, images should contain a homogeneous background that provides high contrast to the object of interest, which is important in facilitating computational segmentation of the plant. White and blue backgrounds are currently supported. Second, a mini color checker (e.g. GretagMacBeth Mini Color Checker) should be placed completely in the field of view. This serves as a reference for automatically standardizing the image in terms of color and scale, which is critically important if one wishes to compare expression from images taken at different focal distances with different cameras and in different lighting conditions. Third, images must be free of glare or shadows to avoid compromising algorithm performance. A more detailed description of the imaging requirements can be found in the software user’s manual ( http://PhenomicsWorld.org/PhenoPhyte/).

Following these imaging recommendations has two important benefits. First, capturing phenotypic expression with images allows experimental data to be saved for future use. New and different analyses, that may become possible as more sophisticated and accurate processing algorithms are developed, can be applied to the stored data. Second, the ability to standardize images from different sources makes it possible to combine or compare phenotypic data from disparate experiments. This enables large-scale analyses to be performed and provides a means for the research community to combine data for more comprehensive analysis of the effects of environmental conditions, treatments, and genotypes.

### Benchmarking of PhenoPhyte

To validate our approach and ensure the accuracy of our algorithms, we conducted a series of experiments to benchmark PhenoPhyte. We compared the area of detached, untreated *Brassica rapa* leaves using PhenoPhyte with those using a leaf area meter and ImageJ, and these experiments showed nearly identical results with R^2^ = 0.979 and R^2^ = 0.996 for the leaf area meter and ImageJ, respectively (see Additional file 1). We compared herbivory on *Arabidopsis thaliana* rosettes using PhenoPhyte with ImageJ and the leaf damage estimation method. We again obtained strong correlations with ImageJ (R^2^ = 0.995 for undamaged rosette area and R^2^ = 0.948 for herbivory (see Additional file 1), but noted visual estimation errors and inconsistencies with the human estimation method. See the supplement for a full description of the benchmarking experiments and a detailed comparison and discussion of the differences in leaf damage scoring between the automated and manual approaches.

### Examples of applications

PhenoPhyte was designed to facilitate measurement of plant area, growth, and herbivory in a number of settings and species and here we illustrate many of them. The section ends with a different and more complicated example application (disease resistance) that is not supported by Phenotype, which demonstrates the flexibility and broadness of this application. See the Additional file 1 for full details on image acquisition and processing and the experimental setup of each example application.

#### Measuring growth in *Arabidopsis thaliana*

By repeatedly and nondestructively imaging Arabidopsis rosettes over time, the software can provide accurate measurements of growth.

**Figure 1:**
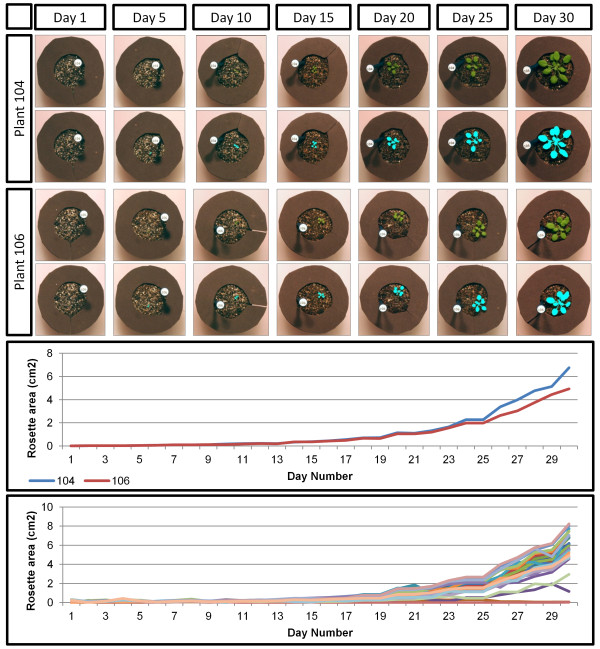
**Example results from the growth experiment.** The top panel includes normalized and processed images for two plants at five-day intervals. The middle panel shows the growth curves for those two example plants, showing the ability to distinguish between individual curves within genotypes. The bottom panel shows the growth curves for all 60 plants in the experiment.

To illustrate, 30 Arabidopsis plants were imaged (6 plants per image) once daily for 30 days. The amount of plant growth per day and the change in plant area over the one-month study are illustrated in Figure 1. The algorithm was able to detect plants as small as 0.004 cm^2^, and detect and quantify slight variations in the growth curves of individual plants. This sensitivity of growth analysis can be useful in identifying and studying more subtle factors (both genetic and environmental) that affect plant growth.

There are two major advantages to the ability to accurately measure growth computationally. First, because traditional means of estimating growth (rubrics, manual estimation, etc.) are slow, laborious, and error-prone and more advanced methods are typically expensive, this method provides a less expensive option for high-throughput, accurate growth measurements to be made. Second, the ability to measure and model individual plants’ growth rates facilitates the adjustment of time-lapse measurements to account for growth of the plant during an experiment.

#### Measuring herbivory in *Arabidopsis thaliana*

By imaging plants before and after insect feeding, the software can provide accurate measurements of leaf herbivory. Plant traits known or hypothesized to influence insect feeding are often assessed by experiments in which insects are allowed to feed on different plant genotypes and the amount of plant matter consumed is used as an indicator of the presence or lack of chemical defense mechanisms.

To demonstrate, 30 *Arabidopsis thaliana* rosettes were exposed to cabbage butterfly caterpillars (*Pieris rapae* L. Pieridae) in cages and 30 plants served as insect-free controls. Images were taken before the introduction of the caterpillar and after feeding concluded 2 hr later.

**Figure 2:**
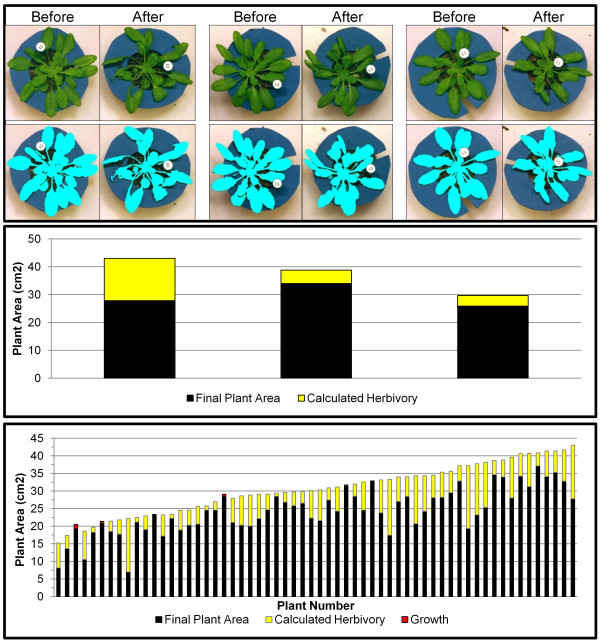
***Arabidopsis thaliana *herbivory results.** The top panel shows before and after images for three plants from the experiment, as well as the processing results underneath. The middle panel illustrates the change in plant area between before and after images with the total height representing the initial area, the black fill representing final plant area, and the yellow fill indicating the amount of herbivory. The bottom panel shows these same results for the full experiment. Red fill indicates increases in plant area between before and after images.

The full results are shown in Figure 2. Original and processed images for three plants, before and after feeding, are shown in the top panel. In the middle panel, the total height of the bars in the graph represents the initial plant matter for each rosette as calculated by the software. The black fill indicates the final plant area, and the yellow fill shows the difference between the initial and final plant area, i.e., the measurement of herbivory. In this case, a simple difference of the plant area before and after insect feeding was used to calculate the leaf area removed by the caterpillar. All but three plants experienced damage from caterpillar feeding as measured by leaf area loss. In three plants (#3, #6, and #20), there were small increases in plant area (denoted by the red “growth” bar fill in the bottom panel), indicating either no caterpillar feeding occurred or little enough feeding that it was compensated for by the small amount of plant growth which occurs in 2 hr.

#### Measuring herbivory in *Brassica rapa*

PhenoPhyte can also measure these traits and phenotypes in other species with similarly flat rosettes or by using detached leaves. In this example, cabbage leaves (*Brassica rapa*) were detached before the experiment began, and images were taken both before and after caterpillar feeding (2 hr).

**Figure 3:**
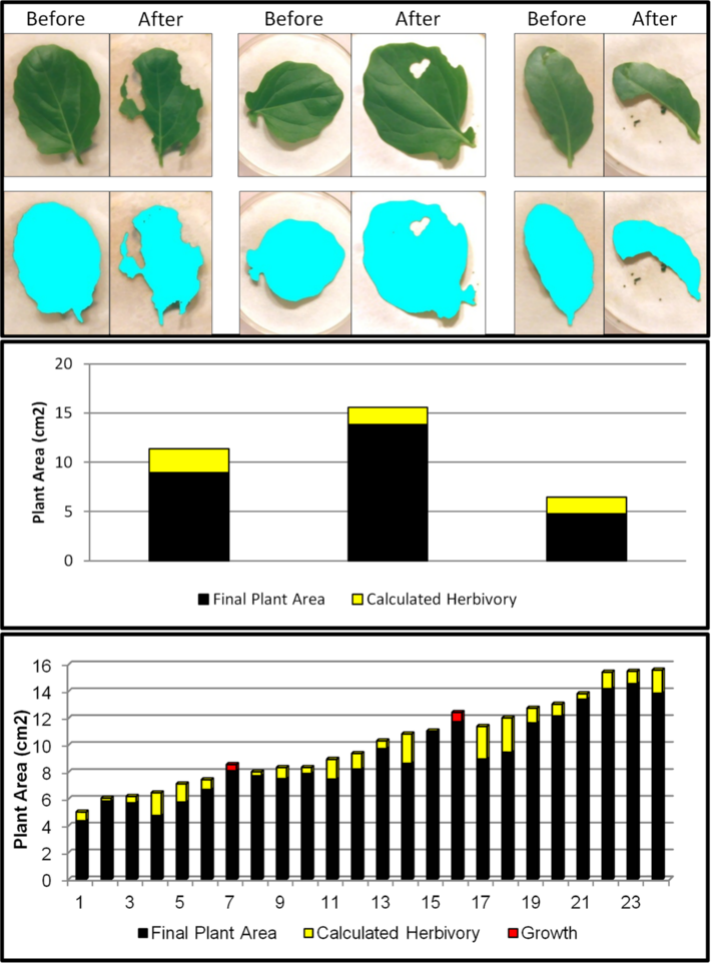
***Brassica rapa *herbivory results.** The top panel shows before and after images for three leaves from the experiment, as well as the processing results underneath. The middle panel illustrates the change in leaf area between before and after images with the total height representing the initial area, the black fill representing final plant area, and the yellow fill indicating the amount of herbivory. The bottom panel shows these same results for the full experiment. Red fill indicates increases in left area between before and after images.

The results are shown in Figure 3. The identification of plant matter is shown in the top panel for three leaves before and after caterpillar feeding. The middle panel again visually quantifies the difference in visible plant area between the before and after images (see Section 2.4.2 for a full description of the meaning of each color). All but two plants experienced damage from caterpillar feeding. Two plants (#7 and #16) had very slight increases in plant area, which resulted from the combination of no observable caterpillar feeding and differences in leaf curvature between images. In this experiment, since the plants were all of the same genotype and grown under identical conditions, variation in the amount of herbivory largely reflects the biological variation in feeding among individual caterpillars.

#### Measuring herbivory with choice assays in *Arabidopsis thaliana*

Uniquely, PhenoPhyte also supports the analysis of herbivore choice assays, in which two different genotypes or treatments are present in one pot, separated by a marker. Choice assays can provide a better estimate of plant resistance to insects because they reflect insect feeding preferences not measured by no-choice assays.

**Figure 4:**
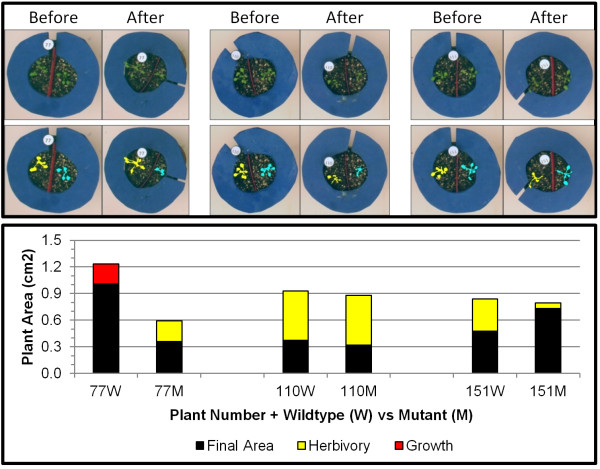
**Examples of choice assay herbivory results for *Arabidopsis thaliana*.** The top panel shows before and after images for three pots from the experiment, as well as the processing results underneath. The middle panel demonstrates plant areas both before and after feeding for the (left) wildtype and (right) mutant plants in each pot.

**Figure 5:**
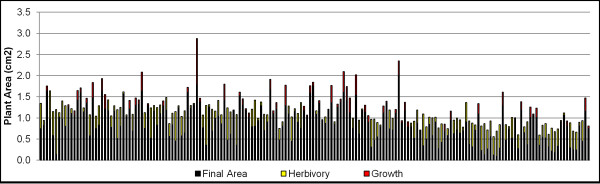
**All results for the choice assay herbivory experiment for *Arabidopsis thaliana.***

To illustrate, we used 120 pots, each containing two small Arabidopsis rosettes: one wild-type and one mutant, and let first instar beet armyworm caterpillars feed on them for 15hr. Figure 4 shows three examples from this experiment. A plot of the areas of the (left) wildtype and (right) mutant plant of each example is shown in the middle panel. The yellow fill indicates the amount of herbivory, whereas the red fill indicates growth between initial and final images. Figure 5 then shows this same display for all the plants in the experiment. In this case, the wild type and mutant genotypes did not differ in size and the caterpillars had a 1.8-fold higher preference for wild type plants (p<0.0058, Wilcoxon Two-Sample Test).

#### Measuring disease resistance to Southern Leaf Blight in *Zea mays*

This technique generalizes to more complicated phenotypes as well. For example, we have developed a separate module, not currently available in PhenoPhyte (though source code can be provided by request), for measuring necrosis and/or lesion burden in leaves. While this has been specifically used here for quantifying disease resistance or susceptibility in maize, many plant bacteria, viruses, and pathogens cause browning, chlorotic spots, and necrotic lesions [18-21], and this would be applicable for any disease that induces necrotic areas or causes clear pigmentation changes in the leaf.

Maize plants from a B73 x Gaspe introgression library that had been infected by the fungus *Cochliobolus heterostrophus* were imaged. The top two ear leaves from two representative plants from each of ~400 rows were nondestructively imaged in the field, for a total of ~1600 images. These leaves were chosen for imaging as they corresponded to the leaves of the plant that were denoted as most important in differentiating resistance levels, according to an infection scoring rubric commonly used with this disease, as in [22] except inverted.

**Figure 6:**
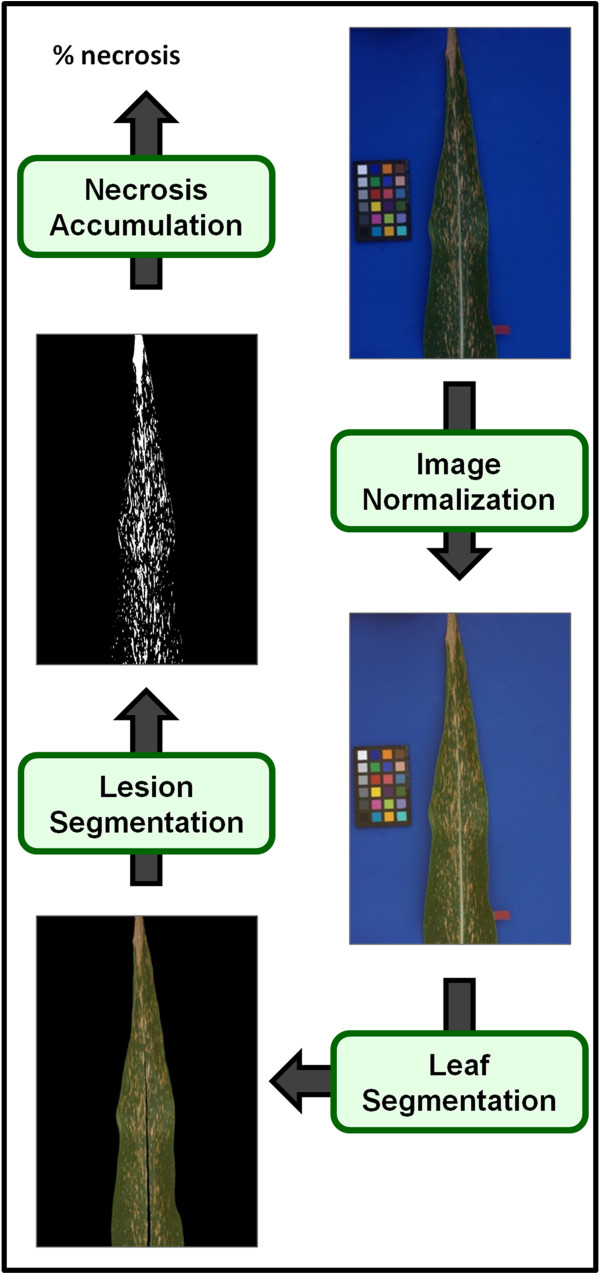
**Processing pipeline for the amount of necrosis in maize leaves.** This measure is used as a proxy in this paper for disease resistance to Southern Leaf Blight.

Automatic processing of these images (as seen in Figure 6) was performed to quantify the infection severity by determining the amount of leaf covered by necrotic lesions. See the Additional file 1 for a detailed comparison of these automatic scores to those manually assigned in the field using the infection scoring scale.

### Biological impacts

Our approach to digital phenotyping has broad biological and horticultural applications It can help researchers answer important scientific questions about the effect of a treatment, genotype, or environment condition on plant growth or susceptibility to insects or pathogens.

Because our approach normalizes the color of each digital image as part of the automated computerized processing, other differences in pigmentation between tissue types could be detected and quantified with relative ease using Phenophyte. This includes the induction of anthocyanins in response to drought, cold, light, or hormone application [23] and the loss of pigmentation due to nutrient deficiency and disease.

The automation of Phenophyte means that measurements can be effortlessly calculated from large sample sizes, which allows one to quantify and account for the large biological variation caused by phenotypic plasticity frequently found even within the same genotype [24-26]. Furthermore, the image normalization of Phenophyte allows comparison among experiments done under different lighting conditions in different labs, and preserves results in a digital format that can be later reused for analysis.

### Limitations

The PhenoPhyte software package does have a few limitations. First, phenotype images must adhere to the imaging requirements because deviations may cause poor quality results and sometimes even an inability for images to be processed by the software. Characteristics known to degrade results are heavy shadows in the image, glare on the leaf surface, overlapping of plants, and a non-uniform image background. Second, manual perusal through the imaging results is highly recommended to ensure high quality segmentation and thus high quality area measurements. This can be potentially time-consuming, especially if the initial parameters for the experiment were far from optimal; fortunately, this can be minimized by careful selection of parameters and early preview of results. Third, the user is assumed to have a basic knowledge of the hue, saturation, value (HSV) color space in order to fine-tune plant identification thresholds; these concepts are explained in the PhenoPhyte user manual. Fourth, although the two-dimensional phenotyping we describe here is useful for a broad range of applications, it is not intended for three-dimensional measurements. Imaging from multiple angles to produce a 3D reconstruction of the leaf (as with the LemnaTec 3D Scananalyzer products, see http://www.lemnatec.com) is one solution; however, the question of how to quickly and reliably do this imaging outside a “controlled environment” remains. Finally, even though processing of phenotype images is quite fast, phenotype capture in the field (especially in experiments with large populations and multiple repetitions) can be much more time consuming than scoring with a rubric. Methods for more automated capture of phenotypes *in the field*, perhaps through advanced robotics, still need to be developed for high throughput imaging in the field. In the case of area, growth, and herbivory however, imaging sets, even large sets, of pots or leaves can be processed faster by PhenoPhyte than by scoring visually.

## Conclusions

In this paper, we introduce PhenoPhyte, a new web-based software application, for fast and accurate measurement of select two-dimensional traits from plant images. The uniqueness of this software is in its ability (1) to process image sequences, track objects across images, and report trait changes over time, (2) to process choice assay experiments, and (3) to automatically correct for variations in lighting and scale, which permits imaging in a variety of locations including the lab, greenhouse, and field and facilitates the comparison or coalescence of data from disparate experiments. To demonstrate the versatility of this software, several example applications were presented that show PhenoPhyte’s ability to measure a variety of visual plant phenotypes in multiple species.

## Methods

### Image acquisition

Images were captured with a Canon Rebel XT camera using a fixed-length 50 mm lens, though any digital camera with adequate resolution would suffice. The camera is used in conjunction with an inexpensive custom-built imaging apparatus that allows mounting of the camera in such a way that the focal line of the camera is orthogonal to the center of the imaging area, which is simply a rectangular base covered with a solid high-contrast color. Before imaging, the height of the camera is adjusted so that only the background is in the field of view (see Additional file 1). The interested reader is directed to [27] for details regarding construction of such an apparatus.

### Specimen placement

Multiple specimens (pots or leaves) may be captured in a single image, though special care must be taken with time series or before/after images to ensure that specimens are in the same relative position as the algorithms use this information to link specimens between images. It is important to either use pots that are not green (e.g., terracotta) or cover the pot edge for photographs because the green pot rim can interfere with plant tissue detection when leaves reach or overlap the pot edge. Removable pot rim covers or collars are easily made from brown or blue craft foam and inserted on the pot rims underneath the leaves for photographs. Most of our experiments used a setup in which a white, hard plastic slab was crafted with six slots for pots and a dedicated position for the color checker. The algorithms, however, are designed to handle up to 254 clearly separated specimens in an image, and the color checker can be detected in any position and orientation in the image.

### Image processing

A GretagMacbeth Mini Color Checker is placed on the background in each image for normalization purposes. A custom suite of algorithms, written using the C language in conjunction with the OpenCV open-source computer vision library, is used to process the images in each experiment.

**Figure 7:**
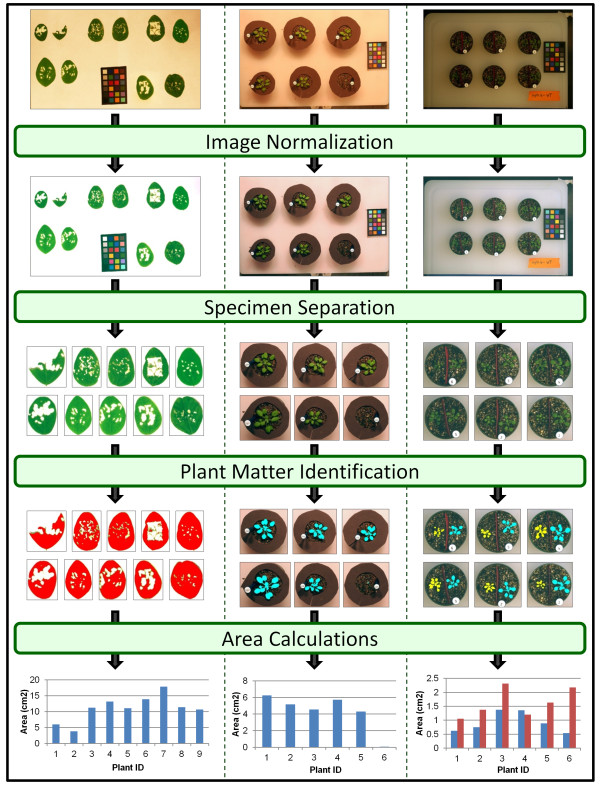
Processing pipeline for measuring area, herbivory, and growth from leaves and rosettes.

The processing pipeline, which includes four major steps, is demonstrated in Figure 7 with an image example from three of the experiments. The first step is that of image normalization. This algorithm proceeds by (a) identifying the color checker in the image, (b) orienting and mapping color wells, and (c) calculating and applying appropriate color channel transformations. Identification of the color checker is accomplished using Sobel edge detectors and Otsu thresholding [28] to maintain heavy edges in the image, 8-neighbor connected components to group edges into objects, followed by the application of a set of heuristics (including component shape, relative size, proximity, and color distribution) to correctly identify those objects that correspond to colors wells in the color checker [17]. After objects have been successfully extracted, they must be correctly mapped to the corresponding color well. This is facilitated by first utilizing relative object positioning to calculate the angular orientation of the color checker. Then, a search is conducted to find the best correspondence of objects to color wells based on object positions and colors (represented by the median RGB colors), with the spatial relationships among objects used to prune the search space. This search is conducted in such a way as to permit successful identification and mapping of the color checker even in cases where significant portions of the color checked are occluded. Once the wells have been mapped, an appropriate transformation matrix is computed by performing a simple linear regression on the pairing of representative object color to true color well values. This matrix is then applied globally to the entire image for color normalization. Size normalization is also attained by first rotating the color checker to align it to the standard x- and y-axes and then computing a scale factor by utilizing the median pixel size of each object and 1 x 1 cm true size of each color well.

The second step in the pipeline is an algorithm to separate specimens in the image, i.e. partition the image into individual pots, leaves, etc. This algorithm begins by converting the image to the HSV color space. Otsu thresholds [28] are computed on both the saturation and value channels, as objects of interest in the image should have both a lower saturation and a higher value than the white background. The threshold results are merged together to ensure remaining pixels meet both saturation and value criteria. The remaining image is partitioned into objects using connected components [17]. Objects in the image are then numbered by traveling in approximate rows (left to right) from the top to the bottom of the image.

Following specimen separation is a user-assisted algorithm for identifying plant matter. In this step, color thresholds are utilized to highlight the desired portion of the plant. The default values correspond to a general green color, which is of broad use for leaves of many plants; however, users have the ability to adjust these controls to attain more fine tuned results. This includes small adjustments, like modifying the green hue to match the plants in the current experiment, or larger changes to isolate, for example, specific aspects of the plant’s appearance (e.g. a change to a purple hue would allow the detection of anthocyanin or a change to a more yellowish color might allow the user to pick up areas of reduced chlorophyll).

The final step in the processing pipeline is the calculation of plant area, herbivory, and growth values. First, the isolated pixels from the plant matter identification step are accumulated as pixel counts. Using the scale factor computed during image normalization, these counts are converted to plant areas in physical units (cm^2^) using the equation below.

(1) $$area\;CM=\frac{pixel\;Count}{{\left(scale\;Factor*100\right)}^{2}}$$

If herbivory or growth values are desired, they can then be computed as the difference in normalized plant areas between the before and after images.

## Competing interests

The authors have no competing interests to declare.

## Authors’ contributions

JG designed and implemented PhenoPhyte, helped in analyzing experiments, and drafted the manuscript. HA provided the biological background and insight to make PhenoPhyte useful for biologists, helped design and coordinate the experiments, and helped draft the manuscript. ER also provided plant science expertise, helped design and analyze experiments, and aided in drafting the manuscript. JH aided in the design of PhenoPhyte, provided technical expertise, and helped draft the manuscript. JC participated in the implementation of PhenoPhyte, provided technical expertise, and critically reviewed the manuscript. PBK provided expertise in corn blight biology and imaging, helped design and analyze those experiments, and aided in drafting the manuscript. CRS oversaw the design of PhenoPhyte and the experiments and helped draft and review the manuscript. All authors read and approved the final manuscript.

## Supplementary Material

Additional file 1 Supplement Word document named Supplement1. Click here for file
