# Supplementary material for: PhenoPhyte: a flexible affordable method to quantify 2D phenotypes from imagery
Source: Plant Methods. 2012 Nov 6;8:45. doi: 10.1186/1746-4811-8-45 (PMC3546069; doi:10.1186/1746-4811-8-45)
Supplement: Additional file 1 — Supplement Word document named Supplement1. [file 1746-4811-8-45-S1.docx]

# RESULTS AND DISCUSSION

## Imaging Protocol

We have developed a protocol for image capture of phenotypes that supports robust and flexible phenotype expression quantification via computer vision and image processing algorithms. The protocol is described in detail in the PhenoPhyte manual (<http://PhenomicsWorld.org/PhenoPhyte/PhenoPhyte_UserManual.pdf>) and summarized below. There are four main components to the protocol, and though relatively simple, they all are critically important to the success and accuracy of our automated phenotyping approach.

The first component is the use of a homogeneous background in the image that provides high contrast to the object of interest. This is important to facilitate accurate computational segmentation or identification of the plant area being imaged. Without this, it can be very difficult for computer programs or the human eye to automatically separate the leaf or plant in the image from other objects, especially when images are taken in field conditions where plants are grown closely together (see Figure S1 for an illustration). With good identification of the plant area of interest, computer algorithms can be more accurate, objective, and consistent in quantifying phenotype expression than humans, which in turn eliminates, or at least minimizes, the need to rely on human memory, perception, and consistency in measuring phenotypes.


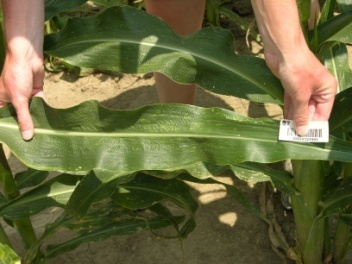

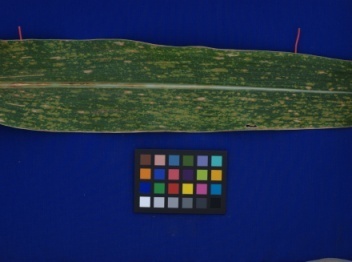


*Figure S1: Example images of maize leaves taken in the field with (A) the image on the left taken without our protocol and (B) the image on the right taken using our imaging protocol.*

The second component of the protocol is the purposeful placement of a mini color checker (e.g. GretagMacBeth Mini Color Checker) completely within the field of view. These color references are well known in the imaging industry and consist of 24 predefined color wells that can be used to evaluate and adjust image color profiles. Because lighting conditions can vary between images, especially when taking images outdoors, the color checker provides a reference that can be used for normalizing images with respect to color. In addition, the known size dimensions of the color checker provide a size reference that can be utilized for normalizing images with respect to scale. By placing the color checker in the field of view, images taken from different distances with different cameras and in different lighting conditions can be transformed to a common baseline that standardizes colorimetric and size measurements to provide accurate comparisons between phenotype images.

The third component of the protocol is only applicable in specific circumstances (e.g. choice assays) and includes the placement of a thin, straight, red marker between plants to facilitate separation of the plants. This is necessary as plants are grown in close proximity for these experiments, and overlaps of leaves complicates plant separation and may compromise algorithm performance.

The fourth and final part of the protocol involves characteristics of the acquired image. The lens should be pointed approximately orthogonal to the plant or leaf being imaged in order to maximize the amount of leaf visualized. In addition, while different lighting conditions are tolerable, care should be taken to minimize shadows in the image, especially shadows on or near the plant area of interest. Finally, plants should not be photographed in direct sunlight as this tends to create large amounts of glare. The use of a grayscale umbrella to shade the plant during photography in the field is advised.

A major benefit of this protocol is that it facilitates *nondestructive* imaging of phenotypes in the field, lab, or greenhouse. Using this approach, many phenotypes can be imaged and quantified without having to destroy or damage the plant. This permits repeated measurements on the same plant, which can be useful for measuring changes over time.

This imaging protocol also provides several other benefits. The ability to capture phenotype expression using images allows experimental data to be saved for future use. New and different analyses, that may become possible as more sophisticated and accurate processing algorithms are developed, can be applied to the stored data. Furthermore, adherence to the defined imaging protocol, which facilitates both spectral and size normalization of images, can allow phenotypic data from several disparate experiments to be combined or compared. This can enable large-scale analyses to be performed and provides a means for the research community to combine data for more comprehensive analysis of the effects of environmental conditions, treatments, and genotypes.

## Comparison of PhenoPhyte with other methods

To validate our approach and ensure the accuracy of our algorithms, we conducted a series of experiments to compare our approach using PhenoPhyte with popular methods for measuring leaf area and herbivory.

### PhenoPhyte vs Leaf Area Meter and ImageJ: Detached Leaf Areas

We first conducted an experiment to compare leaf area calculations using our method with measurements from a leaf area meter, the accepted standard in quantifying plant area which requires plants or leaves to be harvested prior to measurement, and ImageJ (Abramoff et al., 2004). In total, 24 leaves from Brassica *rapa* (IMB218) plants were imaged individually using the apparatus described in the Methods section and scanned using a leaf area meter (CID 202, CID Instruments, Inc., Camas, Washington). Images were then processed using both PhenoPhyte and ImageJ.

The areas (in cm^2^) produced by PhenoPhyte and the leaf area meter are compared in Figure S2(A). The two methods were found to produce highly similar results (R^2^ = 0.979), and this benchmarking against the leaf area meter demonstrates the accuracy of PhenoPhyte in detached leaf assays. Despite the almost identical results, there are three major differences between the methods. First, the two methods deal with leaf curvature in different manners; the leaf area meter flattens leaves while the inherent curvature of the leaves is retained and reflected in photographic imaging. For inherently non-wavy leaves, the flattening of a leaf *can* result in a more realistic leaf area, as leaf curling is minimized by flattening but may translate to less visible leaf area in a top-down 2D projection of a leaf. Wavy leaves (e.g. many mature maize leaves), however, are difficult for both methods. Because a 2D image does not capture surface curvature, area measurements from an image can underestimate the true area, while attempts to flatten wavy leaves with a leaf area meter can result in distortion of the leaf and areas where the leaf folds in on itself, which also introduce error into the measured leaf area.


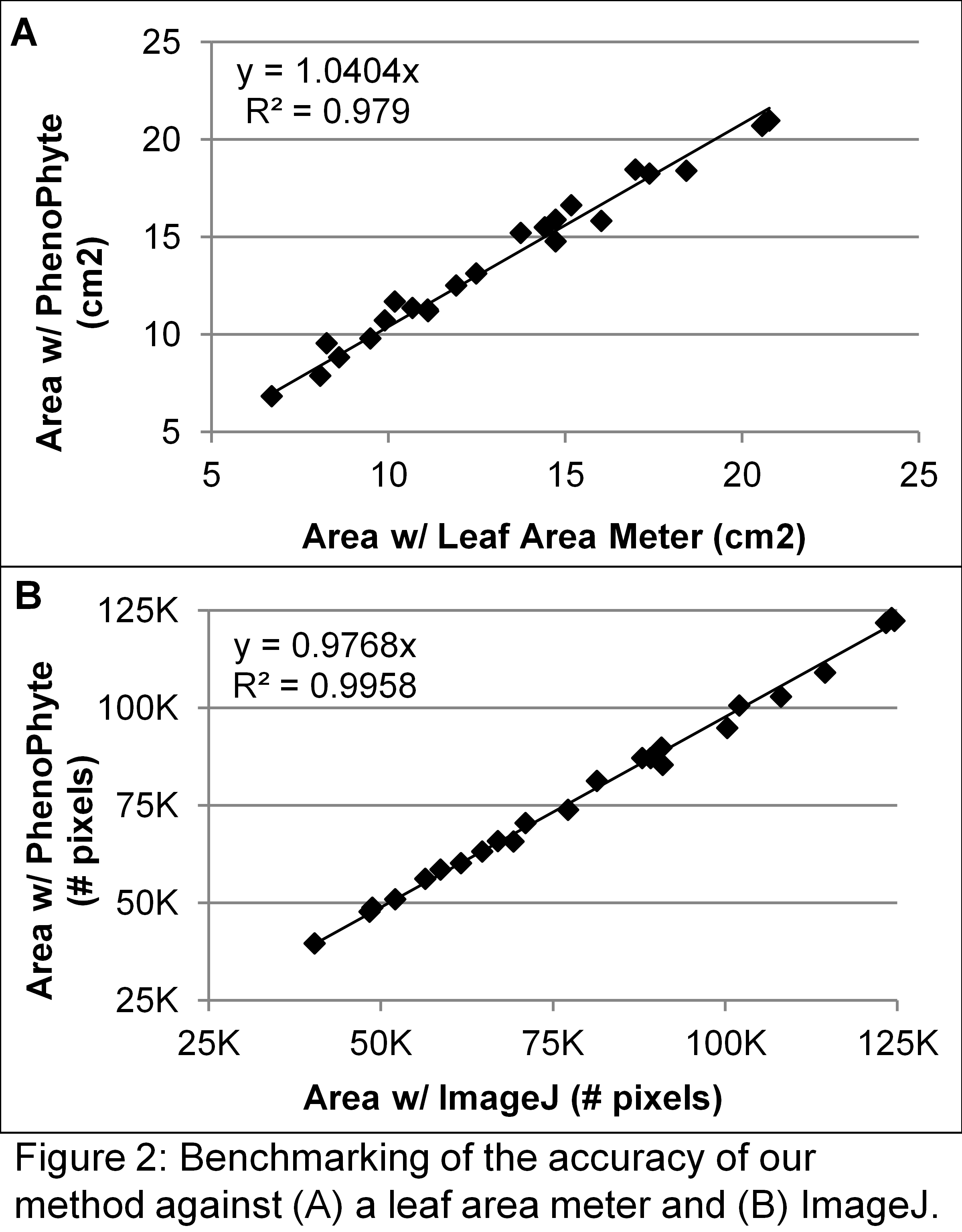


*Figure S2: Benchmarking of the accuracy of our method against (A) a leaf area meter and (B) ImageJ.*

Second, most leaf area meter models require harvesting of the leaf for measurement, whereas imaging by PhenoPhyte can be nondestructive and done in any setting (field, lab, greenhouse, etc). Last, PhenoPhyte imaging is more versatile in that it can be used for measuring whole plant area in rosettes, where these situations are not appropriate for a leaf area meter.

The comparison of PhenoPhyte and ImageJ is shown in Figure S2(B). In this comparison, as expected, we see high similarity in leaf areas with a slightly higher correlation (R^2^ = 0. 996) than with the leaf area meter. This comparison also benchmarks the accuracy of our algorithms against the established ImageJ program.

Again, despite the similarities in output, there are three major advantages to the use of PhenoPhyte over ImageJ. First, Phenophyte has a built-in image normalization routine that standardizes imagery through color correction. To attain the ImageJ results in Figure S2(B), manual color correction of each image was performed in Photoshop before processing began with ImageJ. Without such a step, lighting and color variations across images would make a general-purpose leaf detection process very difficult to write in ImageJ. Second, the normalization routine that is packaged into PhenoPhyte contains a size reference that allows conversion of pixel areas to cm^2^, whereas ImageJ provides results in number of pixels and, without extra user-supplied information, cannot provide area results in physical units. Last, PhenoPhyte is much easier to use. Whereas automating these processes in ImageJ requires the creation of a macro, which is a nontrivial task for researchers with little to no programming background, the Phenophyte processing pipeline already exists and requires little technical background from users to operate.

### PhenoPhyte versus Human Scoring and ImageJ: Herbivory

## There are many situations in which the use of a leaf area meter is impractical, either because destructive sampling is undesirable or leaves are fragile and prone to damage during transfer to the leaf area meter. There are several methods that use human visual scoring to estimate the leaf area changes caused by insect herbivory on intact plants or detached leaves (http://prometheuswiki.publish.csiro.au/tiki-index.php?page=Determining+herbivory+rate). . One of the most widely used and simplest methods for calculating the extent of herbivory is a leaf damage range estimation (Stotz et al., 2000) that utilizes a scale based on ranges of percentages of leaf area removed (see S Table 1).

| **Score** | **% Leaf Area Removed** |
| --- | --- |
| 0 | [ 0% - 5% ) |
| 1 | [ 5% - 13% ) |
| 2 | [ 13% - 23% ) |
| 3 | [ 23% - 37% ) |
| 4 | [ 37% - 55% ) |
| 5 | [ 55% - 77% ) |
| 6 | [ 77% - 100% ] |

*S Table 1: Scoring rubric for the commonly used leaf damage estimation method.*

## Using a test set of 30 *Arabidopsis thaliana* rosettes, we compared herbivory calculations using PhenoPhyte with the leaf damage estimation method, and then with ImageJ. Images were taken of each pot before and after caterpillar feeding. Images were processed with PhenoPhyte and ImageJ to automatically measure the amount of leaf material in both before and after images. Five biologists were recruited to manually assess herbivory using the leaf damage estimation method based on three kinds of information: the live plants right after herbivory (LIVE after), images of the plants after herbivory (IMAGE after) and images of the plants before and after herbivory (IMAGE before & after).

## We first compared the leaf damage estimation method of the live plants against our automated approach. Figure S3 shows comparisons of these two approaches for each of the five biologists and the average human score. On average (bottom right), the two methods agreed (i.e. when the blue triangle falls within the colored rectangle) in only 20% of the cases (6 out of 30) and differed by at most one scoring category in 73% of the cases (22 out of 30). The remaining 27% showed more significant differences (at least 2 scoring categories) in herbivory measurement. In general, manual scoring tends to underestimate small to moderate amounts of leaf damage, and tends to overestimate large amounts of leaf damage (see Figure S3). The experiment as a whole demonstrates the need for objective, consistent, automated scoring, as the correlation coefficient between the two scoring methods ranged from 0.58 to 0.77 depending on the scorer.

The differences in leaf damage assignments may be attributed to several factors. First, because the human-scoring method does not document the initial state of the plants (while the automated approach does), a mental reconstruction of the original state of the entire plant is required to estimate leaf damage. This can be particularly difficult in cases where large portions of a leaf or entire leaves are consumed or lost. Second, the human-scoring method relies heavily on human perception and consistency. In addition to being able to identify all places where portions of the plant are missing, a scorer must be able to accurately estimate the percentage of plant removed and replicate this accuracy over time. All inconsistencies in reconstruction and assignment of damage classes result in error incorporated into the damage estimation. The automated approach is not without limitation, however. First, the images used in the computer-scoring method can only measure the amount of plant matter visible from a top-down two-dimensional view of the plant. Inherent leaf curvature may cause some underestimation of leaf area. Also, because rosette leaves can overlap, the automatic method


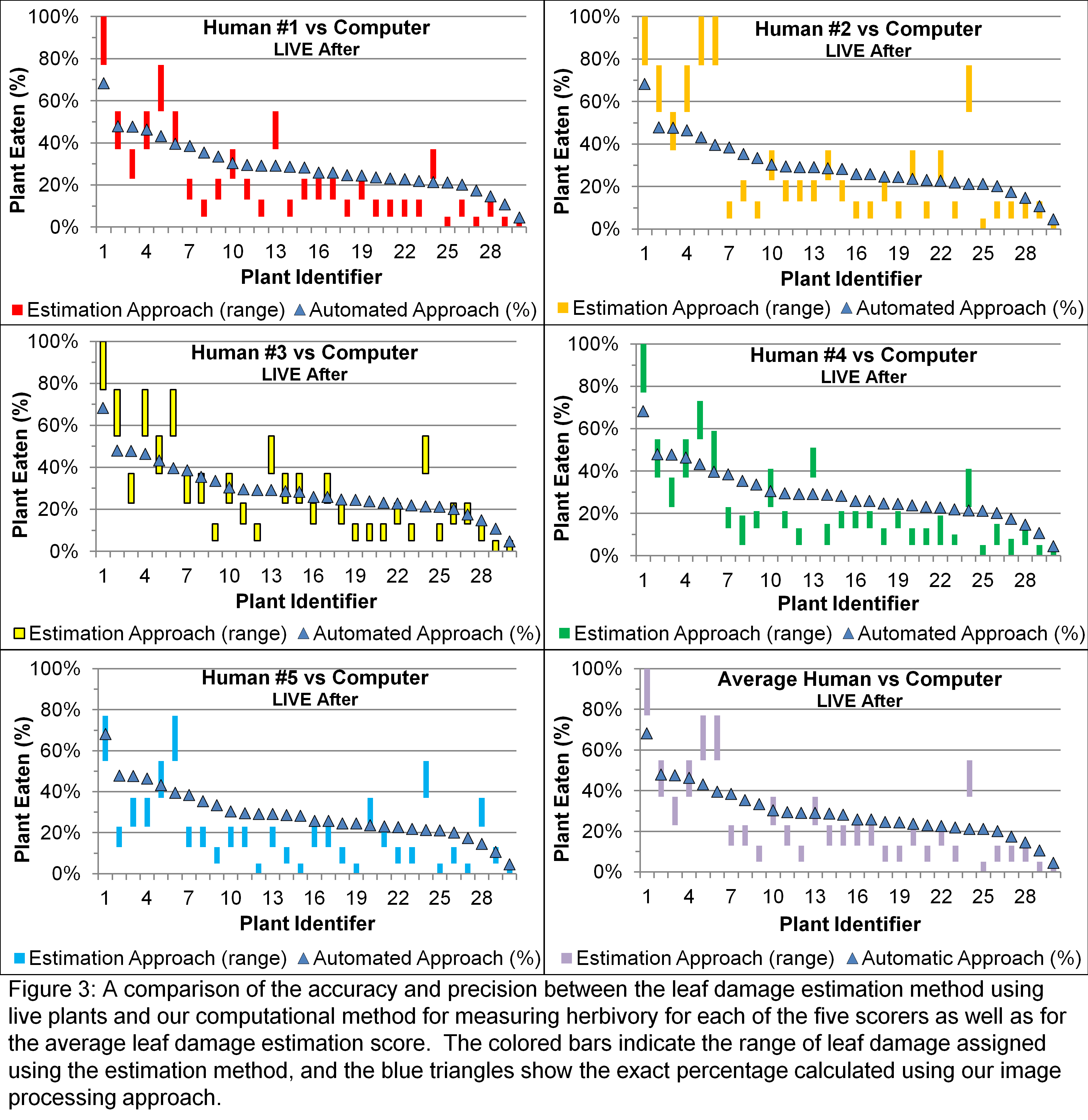
*Figure S3: A comparison of the accuracy and precision between the leaf damage estimation method using live plants and our computational method for measuring herbivory for each of the five scorers as well as for the average leaf damage estimation score. The colored bars indicate the range of leaf damage assigned using the estimation method, and the blue triangles show the exact percentage calculated using our image processing approach.*

cannot measure damage done to “hidden” leaves, although in this experiment the visible leaves were primarily the ones eaten by the herbivores.

The computer-scoring method has additional advantages over the human-based scoring method. First, the information that interests most plant scientists is the absolute amount of plant eaten, not the percentage relative to the original plant area that the human-based scoring provides. The computer-scoring method can calculate an absolute leaf area eaten, which when combined with a sampling of leaf thickness can be used to determine a volume of plant matter consumed.

Second, the human-scoring method relies on arbitrary intervals to score plants, which means that plants with only subtly different leaf damage could end up with different scores. To demonstrate, consider Figure S4, in which the actual percentage from the computer-scoring method is plotted against the score this percentage maps to on the leaf damage estimation scale. In particular, consider the percentages near the cutoff between scores 2 (13% - 23%) and 3 (23% - 37%). The plants that flank this boundary have a measured plant area removed of 22.8% and 23.1%, a difference of only 0.3%, and yet their damage scores are different. This loss of precision introduces noise that may affect phenotype analyses. Because the computer-scoring method can generate more precise percentages, there is no need to partition the percentages into these ranges, and thus this issue is avoided.


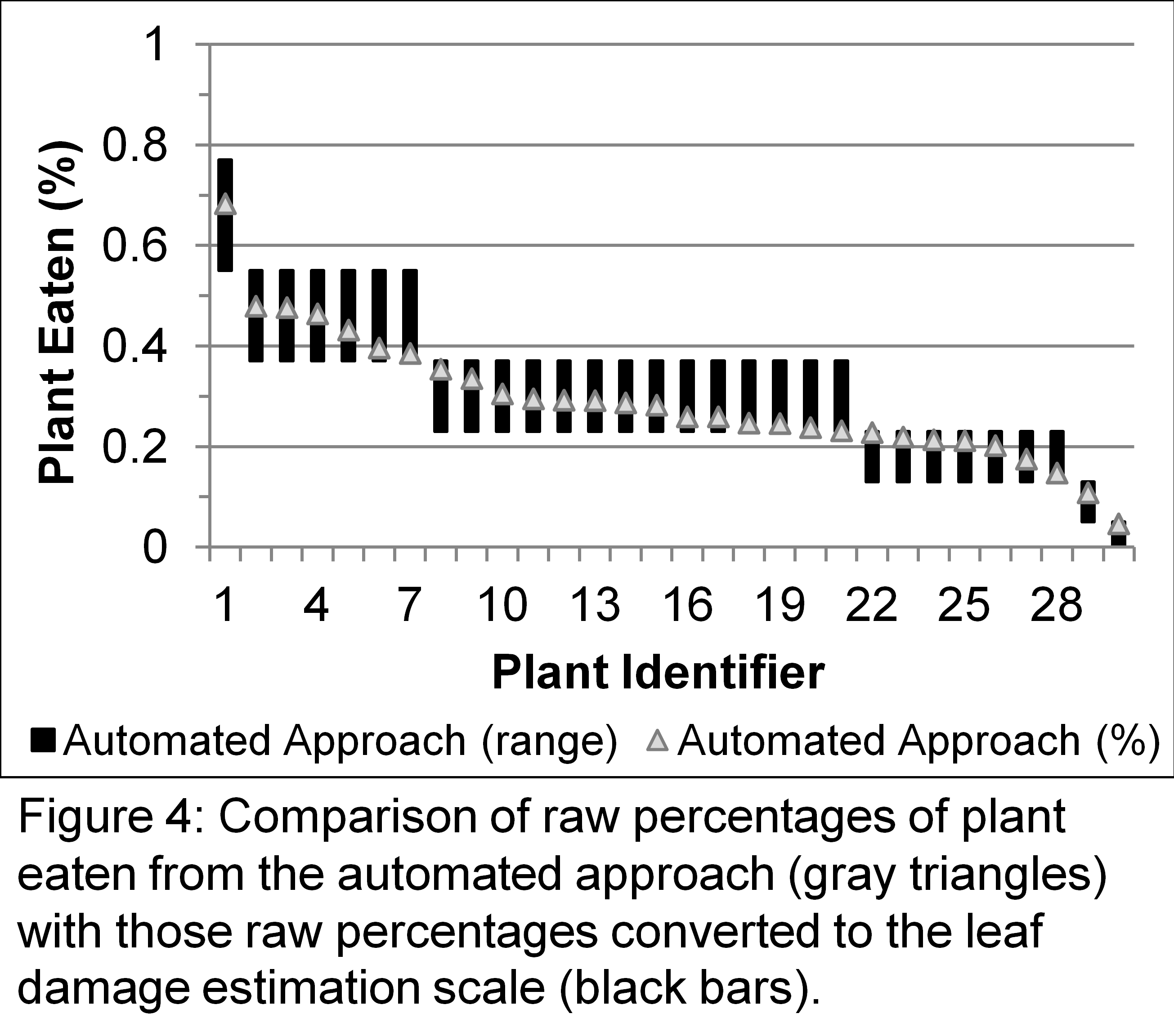


*Figure S4: Comparison of raw percentages of plant eaten from the automated approach (gray triangles) with those raw percentages converted to the leaf damage estimation scale (black bars).*

In addition to scoring of the live plants (LIVE after), we also had each human scorer assign a leaf damage estimation value in two additional ways, by visual inspection of only the “after” images of each plant on a computer screen (IMAGE after), and utilizing both the “before” and “after” images on a computer screen (IMAGE before & after). Each of these rounds of scoring was done independently at least one day apart. The experiment was designed in this manner to not only allow a comparison of the two methods, but also to examine the consistency and accuracy of human estimation on this task.


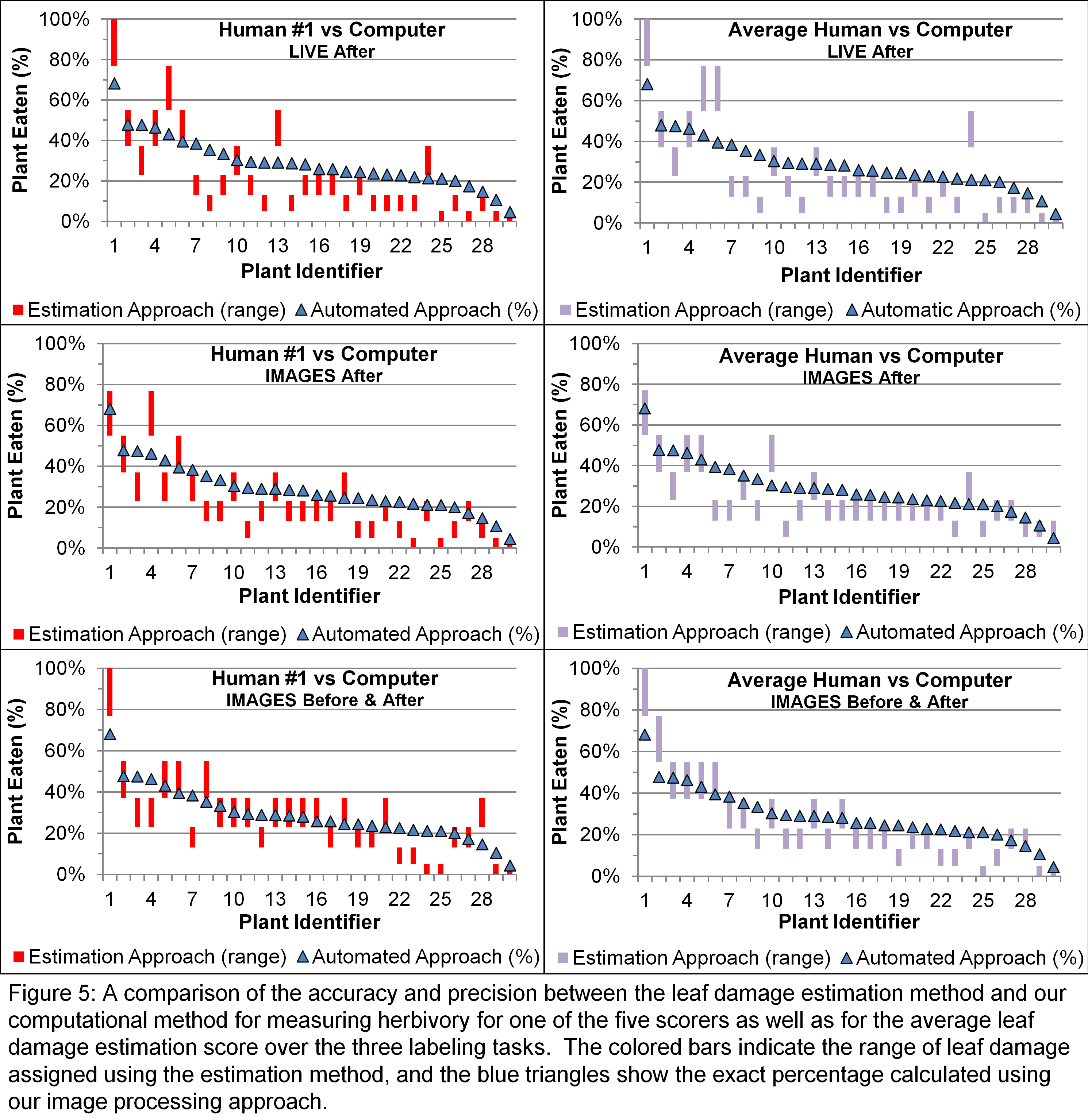


*Figure S5: A comparison of the accuracy and precision between the leaf damage estimation method and our computational method for measuring herbivory for one of the five scorers as well as for the average leaf damage estimation score over the three labeling tasks. The colored bars indicate the range of leaf damage assigned using the estimation method, and the blue triangles show the exact percentage calculated using our image processing approach.*

We then examined the within-scorer differences in leaf damage assignment across the three estimation tasks. The first two tasks require biologists to do a mental reconstruction of the “before” state of the plant for herbivory assessment, whereas the latter two tasks involve scoring based on a 2D projection of the plant.

The results from this experiment show little difference in scoring between the first two tasks, indicating little information difference between viewing the plant itself and viewing a top-down image of the plant; however, there is evidence of improvement in scoring with the third task, the task that requires less mental reconstruction. Across the five scorers, the average number of perfect matches with the automated approach was 22.0% and 25.3% for tasks 1 and 2, respectively, compared to 32.0% for task 3, with this improvement being just outside the 5% significance level (p-value = 0.0695 when comparing task 3 against both tasks 1 and 2 using the Fischer’s Exact Test). Similarly, the average number of times the two methods were separated by at most one scoring category was 66.7% and 69.3% for tasks 1 and 2, respectively, compared to 82.0% for task 3; this is a statistically significant difference (p-value = 0.0016 using the same test as above). Figure S5 shows these same trends visually using plots from an individual scorer (the scorer who over the three tasks best matched the automated scoring) as well as from the average scores for all humans across the three tasks. These results provide further support for the improved accuracy and consistency in using imagery and automated scoring for assessing change in plant area.

We then compared the results produced by PhenoPhyte and ImageJ with these herbivory images. Figure S6(A) shows the relationship between the methods in terms of raw areas for each plant and each timestamp. Again, we see extremely similar, highly correlated areas (R^2^ = 0.995) for individual plants both before and after herbivory. This again shows the accuracy of our approach in measuring leaf area, as compared to ImageJ. Secondly, we plot the herbivory measurements from the two methods against one another (Figure S6(B)). As expected, the correlation values are slightly lower, though still high (R^2^ = 0.948), as herbivory includes the combination of two area measures, each of which may be slightly different from one another.


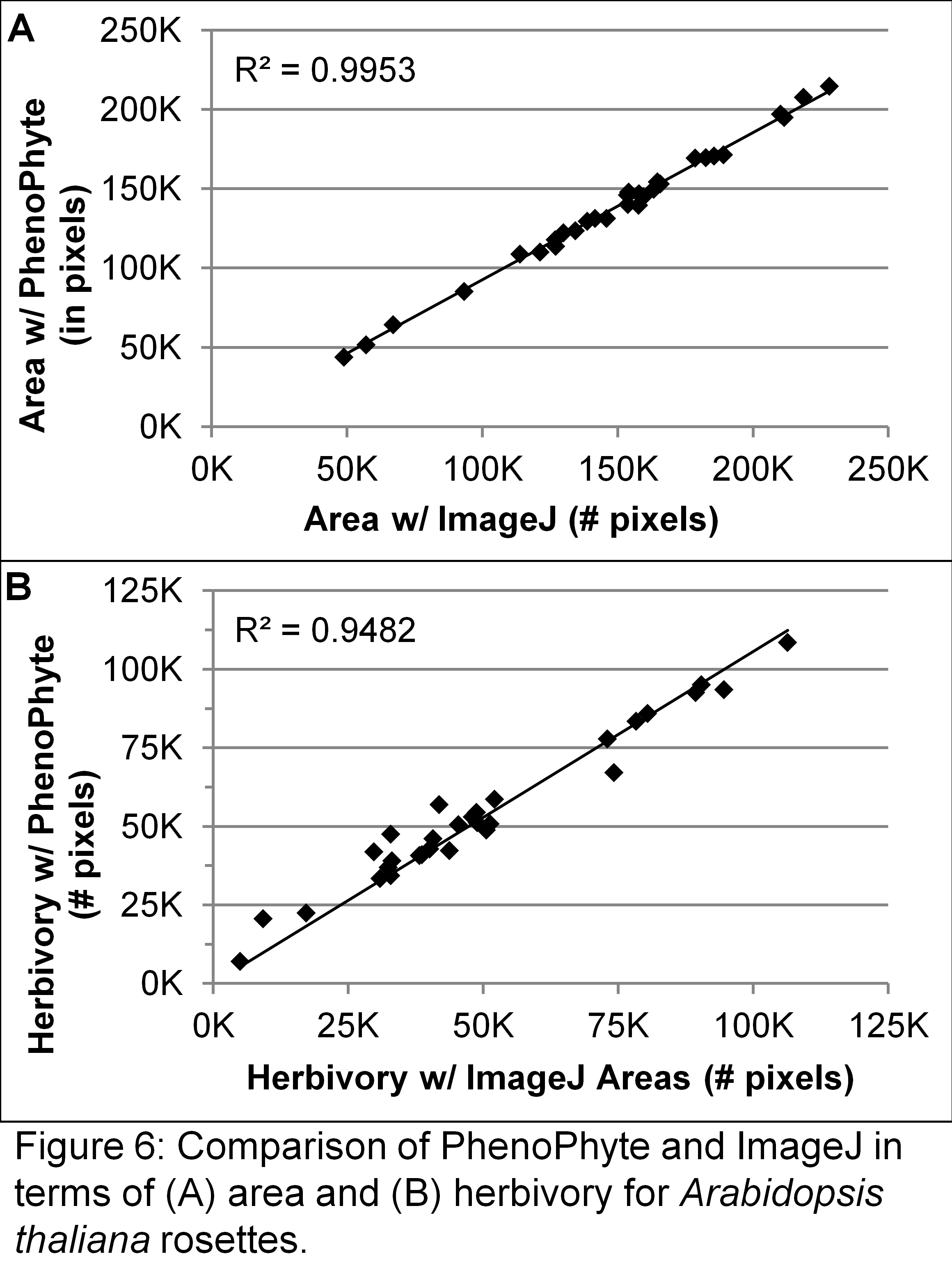


*Figure S6: Comparison of PhenoPhyte and ImageJ in terms of (A) area and (B) herbivory for* Arabidopsis thaliana *rosettes.*

## Examples of applications

Additional details regarding image acquisition and the experimental setup of each example applications are provided here.

### Measuring growth in Arabidopsis thaliana

Brown foam collars were affixed around the pot rims to avoid confusion between the green pots and the plants. Figure 2 (in the main document) contains snapshots of two plants (#104 and #106) over the one-month study and illustrates the different growth patterns of each plant. Brown foam collars were placed between the leaves and the otherwise visible green edge of the pots during photographs to facilitate clean segmentation; this is unnecessary with pots of other colors. Images were taken daily of the plants with image renaming performed appropriately for growth experiment processing with PhenoPhyte.

### Measuring herbivory in Arabidopsis thaliana

This set consisted of the 30 plants in the leaf damage estimation described above and 30 additional control plants. Fourth instar caterpillars of cabbage butterflies (Pieris rapae L. (Pieridae)) were allowed to feed on them for 3 hours. Blue foam collars were affixed around the pot rims to avoid confusion between the green pots and the plants.

For experiments like this one in which there were relatively short time periods of insect feeding and hence little plant growth, it is not necessary to account for plant growth. However, as the time interval between the final and initial images increases, herbivory calculations need to be adjusted to take into account the amount of plant growth, using control plants not exposed to insect feeding.

### Measuring herbivory in Brassica rapa

Cabbage leaves (*Brassica rapa)* were removed from plants and placed individually in petri dishes containing moist filter paper to maintain leaf hydration and prevent wilting. Fourth instar caterpillars of cabbage butterflies (*P. rapae*) were allowed to feed on them for 2 hours. Images of individual leaves in petri dishes were taken before and after caterpillar exposure. Image renaming was performed appropriately for herbivory experiment processing with PhenoPhyte.

### Measuring herbivory in Glycine max

Herbivory by beet armyworm (*Spodoptera exigua* Hübner (Noctuidae)) was measured in soybean as part of a larger study to examine the effects of drought stress and plant genotype on caterpillar feeding. First instar caterpillars were caged for 5 days on leaflets of drought-stressed and control plants on each of two cultivars that had previously been shown to differ in their susceptibility to this insect. Images of leaves at the start of the experiment (before herbivory) were simulated by using Photoshop to individually fill in the ‘missing’ original leaf margins and the holes made by the insect.

### Measuring herbivory with choice assays in Arabidopsis thaliana

As with standard herbivory experiments, images were taken before and after the caterpillar was permitted to feed, with six pots per image. A red marker was positioned between the plants in each pot to comply with the imaging protocol. Brown foam collars were placed on the pot rims to avoid confusion between the green pots and the plants. First instar beet armyworm caterpillars (*Spodoptera exigua* L. (Noctuidae)) were allowed to feed on the plant for 15 hours. Processing was again conducted utilizing the software package, after appropriate image renaming was accomplished.

The experiment can be analyzed to account for two factors: 1) the amount of starting plant area available to the herbivore, and 2) the growth rate of each plant if no herbivore control plants were included in the experiment. In this case, the wild type and mutant genotypes did not differ in size and the caterpillars had a 1.8-fold higher preference for wild type plants (p<0.0058, Wilcoxon Two-Sample Test).

## Measuring disease resistance to Southern Leaf Blight in Zea mays

The software package is specifically designed to measure traits like area, growth, and herbivory by automatically processing and tabulating results; however, the imaging protocol itself is a general-purpose phenotype imaging method that can be used for many other species and can be adapted to measure other and more complicated phenotypic traits. For example, disease resistance can be quantified if the disease causes changes in pigmentation. Here we analyzed the resistance to Southern Leaf Blight using a B73 x Gaspe introgression library, a disease caused by the fungus *Cochliobolus heterostrophus* that causes necrotic lesions to appear on the leaves and can affect yield. Infection severity was first scored manually using a 1 to 9 scale where 9 indicates no phenotypic expression of the disease and 1 indicates death of the plant, as in (Kump et al., 2011) though inverted. Multiple experts scored the introgression library separately. Field scoring was performed by examining the expression of whole plants (all 10-12 plants per row), giving the most importance, however, to the appearance of top two ear leaves. For the automated approach, imaging was performed by selecting two representative plants from each row and nondestructively imaging the top two ear leaves, that portion of the plant most important for differentiating resistance. Automatic processing of these images (as seen in Figure 7 in the main document) was used to compute infection severity.

As we cannot directly measure pathogen growth by examining a leaf image, an indirect measurement was utilized as a proxy for the degree of disease resistance. In particular, the automated approach used the percentage of the leaf covered by necrotic lesions as its phenotype score. For example, plants whose imaged leaves were completely dead were given a score of 100, and those whose leaves were half covered by necrosis were given a score of 50.

Four plant scientists were asked to assess disease resistance both in the field (LIVE) as well as using images of the representative leaves (IMAGES) using the rubric. For each expert,


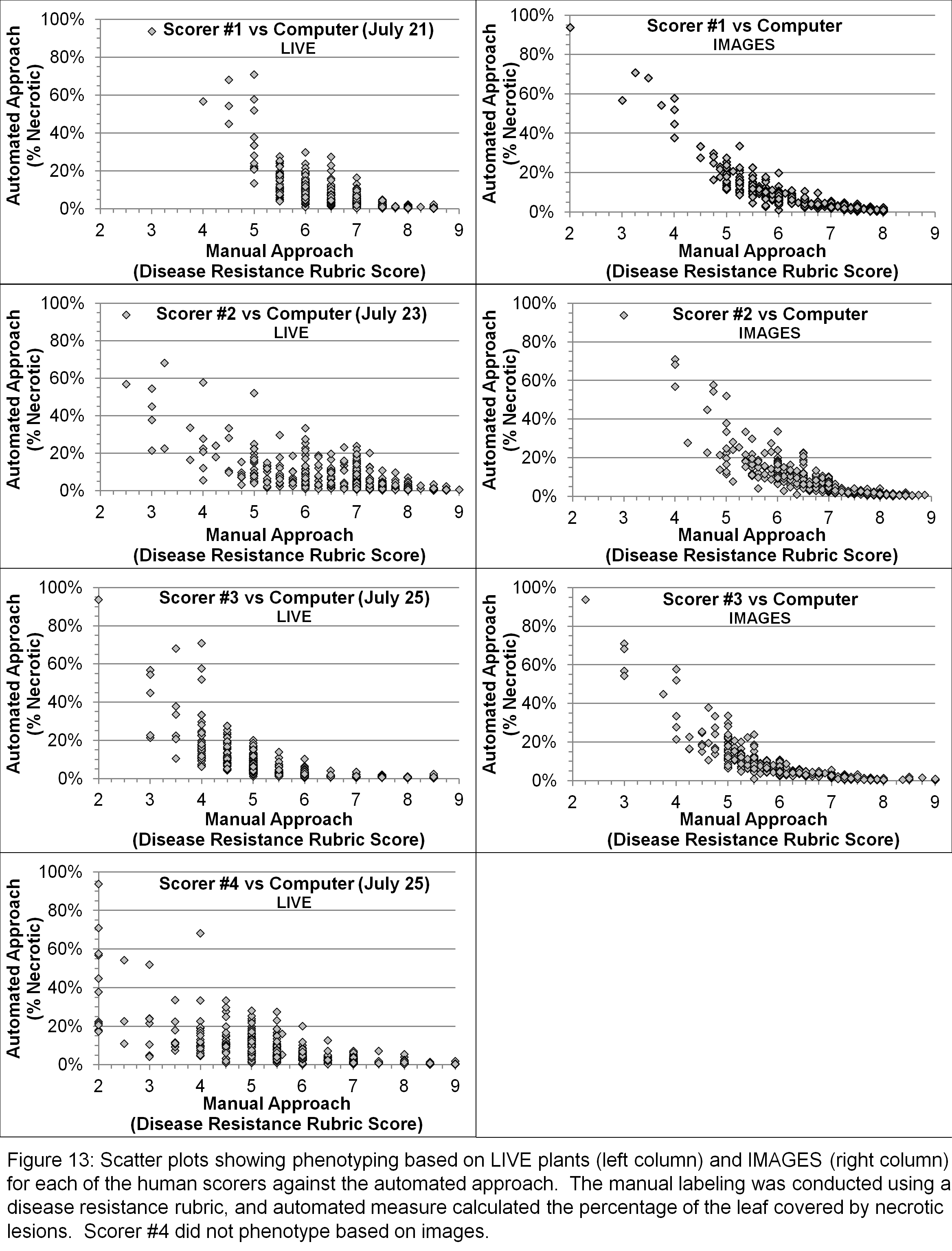


*Figure S8: Scatter plots showing phenotyping based on LIVE plants (left column) and IMAGES (right column) for each of the human scorers against the automated approach. The manual labeling was conducted using a disease resistance rubric, and automated measure calculated the percentage of the leaf covered by necrotic lesions. Scorer #4 did not phenotype based on images.*

phenotyping in the field was completed in a single day; scoring between experts varied over a four-day period with two experts scoring on the same day—imaging was conducted over a three-day period within this timeframe. In general, resistance phenotypes do not appreciably change during this time period.

As before, the human-supplied scores from the field were first compared to the computer scoring. Figure S8 shows scatter plots of the three human scorers against the automated scoring approach for both live plants (left column) and images (right column). When examining the plots from live plants (left column), one first notices the marked overall variation in the plots between scorers as well as the wide range of amounts of necrosis for individual scores. For example, for scorer #1 (top left) and rubric value 5, the amount of necrosis on the top two leaves ranged from 13.5% to 70.9%. These differences may be partially attributed to a number of factors. First, when phenotyping in the field, scientists utilize more information than is captured with imaging: all plants are considered instead of just the two representative plants imaged, and the entire plant is examined versus the top two ear leaves with imaging (though those leaves are the most important, and descriptions of scores 3-7 in the rubric refer only to these leaves). Second, there may be some changes in the phenotype between the times when the plants were scored, though again resistance phenotypes do not appreciably change over this period, and this cannot fully explain the variation as the two scorers who phenotyped on the same day had starkly different patterns. Third, the percent necrosis measure may not be detailed enough


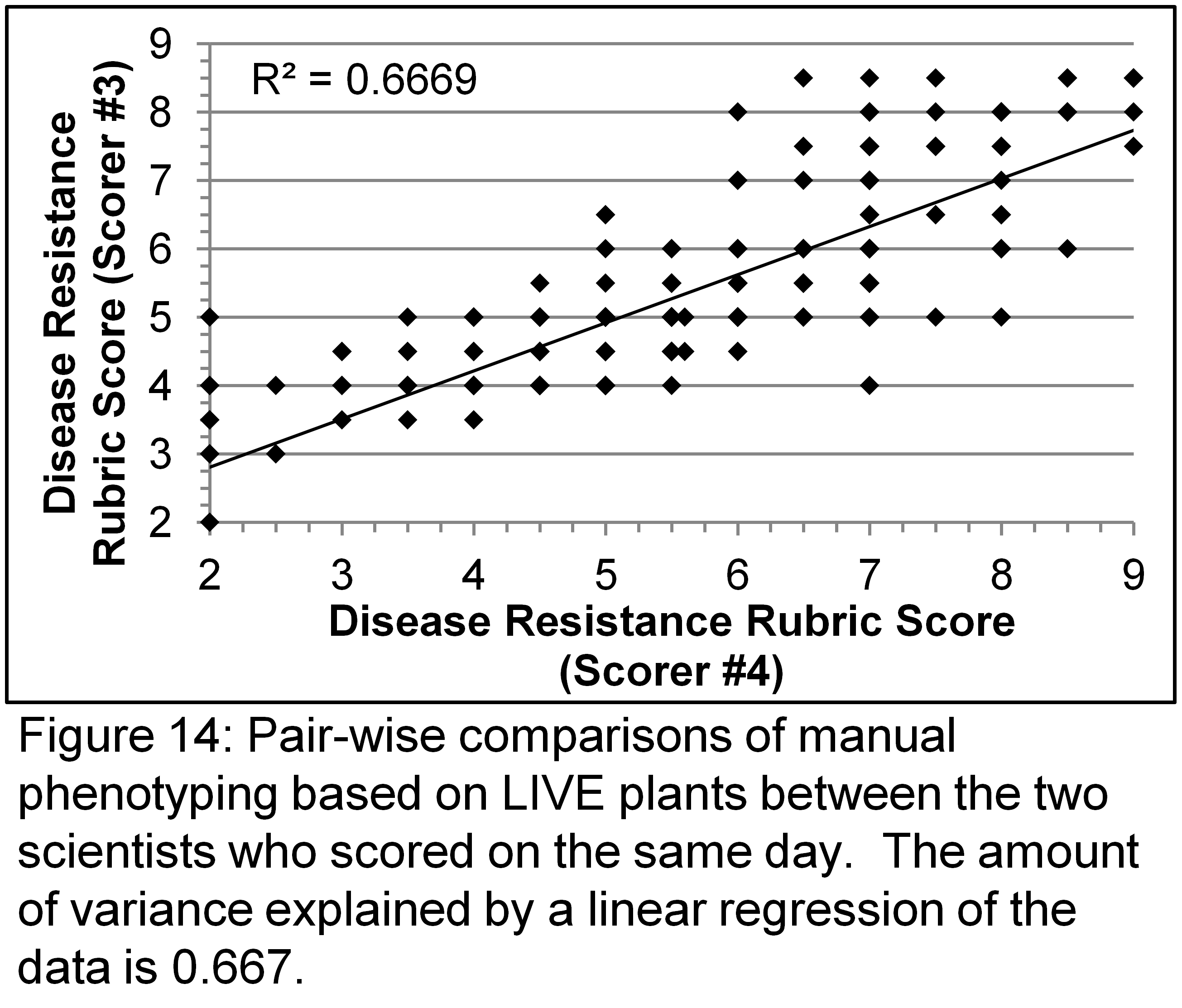


*Figure S9: Pair-wise comparisons of manual phenotyping based on LIVE plants between the two scientists who scored on the same day. The amount of variance explained by a linear regression of the data is 0.667.*

to distinguish between different disease resistance scores as, for example, the sizes of individual lesions on the leaf are lost in the aggregate percent necrosis measure. Last, there may be inconsistencies and inaccuracies in human labeling, which is supported in particular by the variation in plots between the two scientists who scored on the same day (see Figure S9). See Bock et. al. (2010) for additional discussion of sources of phenotyping errors from visual assessment and imagery.


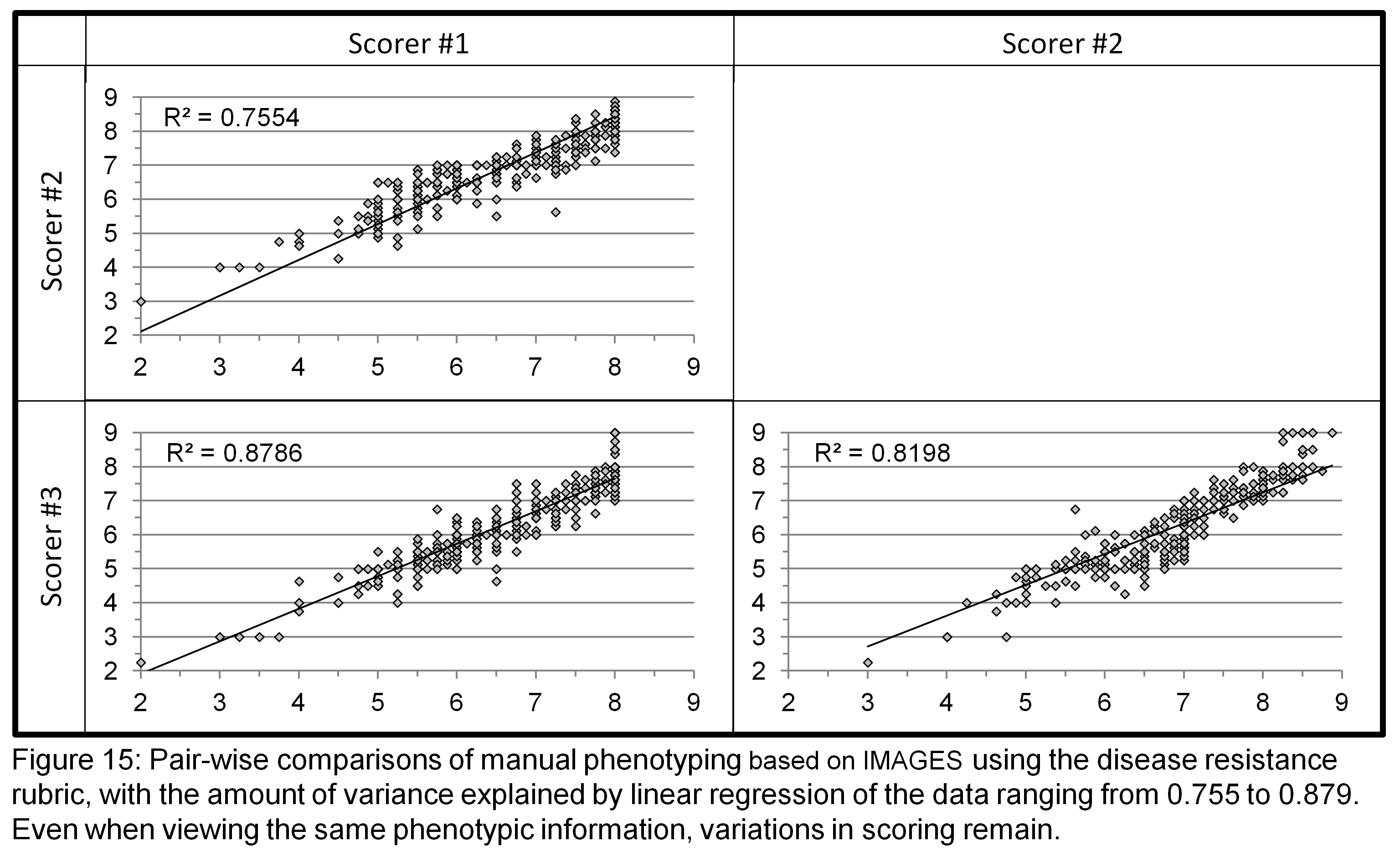


*Figure S10: Pair-wise comparisons of manual phenotyping based on IMAGES using the disease resistance rubric, with the amount of variance explained by linear regression of the data ranging from 0.755 to 0.879. Even when viewing the same phenotypic information, variations in scoring remain.*

Plant scientists also phenotyped the plants based on images (right column). This was done to further investigate between-scorer variation while eliminating variation due to information not included in the captured images and changes in plant appearance due to phenotyping timing. It should be noted that only three of the four experts who had previously scored the LIVE plants participated in this portion of the study. As expected, the scatter plots are much more similar when phenotyping is based on the images, though obvious human-related variations remain. To explore this in more depth, Figure S10 shows the pair-wise comparison of manual labeling. As these graphs compare scores on a plant-by-plant basis, they allow better visualization of the variation between scorers. To quantify this variation, a linear regression was performed on each plot, and the amount of variance explained by the regression ranged from 0.688 to 0.879. As with the herbivory experiment earlier, these kinds of variations demonstrate the noise that is introduced in these kinds of manual phenotyping and the need for more objective and consistent scoring using automated approaches.

ABRAMOFF, M. D., MAGELHAES, P. J. & RAM, S. J. 2004. Image Processing with ImageJ. *Biophotonics International,* 11**,** 36-42.

BOCK, C. H., POOLE, G. H., PARKER, P. E. & GOTTWALD, T. R. 2010. Plant Disease Severity Estimated Visually, by Digital Photography and Image Analysis, and by Hyperspectral Imaging. *Critical Reviews in Plant Sciences,* 29**,** 59-107.

KUMP, K. L., BRADBURY, P. J., WISSER, R. J., BUCKLER, E. S., BELCHER, A. R., OROPEZA-ROSAS, M. A., ZWONITZER, J. C., KRESOVICH, S., MCMULLEN, M. D., WARE, D., BALINT-KURTI, P. J. & HOLLAND, J. B. 2011. Genome-wide association study of quantitative resistance to southern leaf blight in the maize nested association mapping population. *Nat Genet,* 43**,** 163-168.

STOTZ, H. U., PITTENDRIGH, B. R., KROYMANN, J., WENIGER, K., FRITSCHE, J., BAUKE, A. & MITCHELL-OLDS, T. 2000. Induced Plant Defense Responses against Chewing Insects. Ethylene Signaling Reduces Resistance of Arabidopsis against Egyptian Cotton Worm But Not Diamondback Moth. *Plant Physiology,* 124**,** 1007-1018.
